# Contrast-enhanced ultrasound tracking of helical propellers with acoustic phase analysis and comparison with color Doppler

**DOI:** 10.1063/5.0097145

**Published:** 2022-08-02

**Authors:** S. Pane, M. Zhang, V. Iacovacci, L. Zhang, A. Menciassi

**Affiliations:** 1 The BioRobotics Institute, Scuola Superiore Sant'Anna, Pisa, Italy; 2 Department of Excellence in Robotics & AI, Scuola Superiore Sant'Anna, Pisa, Italy; 3 Department of Mechanical and Automation Engineering, The Chinese University of Hong Kong, Hong Kong SAR, China

## Abstract

Medical microrobots (MRs) hold the potential to radically transform several interventional procedures. However, to guarantee therapy success when operating in hard-to-reach body districts, a precise and robust imaging strategy is required for monitoring and controlling MRs in real-time. Ultrasound (US) may represent a powerful technology, but MRs' visibility with US needs to be improved, especially when targeting echogenic tissues. In this context, motions of MRs have been exploited to enhance their contrast, e.g., by Doppler imaging. To exploit a more selective contrast-enhancement mechanism, in this study, we analyze in detail the characteristic motions of one of the most widely adopted MR concepts, i.e., the helical propeller, with a particular focus on its interactions with the backscattered US waves. We combine a kinematic analysis of the propeller 3D motion with an US acoustic phase analysis (APA) performed on the raw radio frequency US data in order to improve imaging and tracking in bio-mimicking environments. We validated our US-APA approach in diverse scenarios, aimed at simulating realistic *in vivo* conditions, and compared the results to those obtained with standard US Doppler. Overall, our technique provided a precise and stable feedback to visualize and track helical propellers in echogenic tissues (chicken breast), tissue-mimicking phantoms with bifurcated lumina, and in the presence of different motion disturbances (e.g., physiological flows and tissue motions), where standard Doppler showed poor performance. Furthermore, the proposed US-APA technique allowed for real-time estimation of MR velocity, where standard Doppler failed.

## I. INTRODUCTION

Small scale untethered biomedical robots offer interesting perspectives toward minimally invasive interventions and targeted therapy, thanks to their ability to access and operate in hard-to-reach body districts.^1^ In all the envisioned applications, therapy success is highly dependent on the ability to control and monitor medical microrobots (MRs) from outside the body.^2^ Several strategies have been proposed to remotely actuate untethered MRs, exploiting different external stimuli including electromagnetic radiation (e.g., light, x rays), chemical reactions, ultrasound, and magnetic fields.^3^ Among these, the latter is arguably the most widely adopted due to its intrinsic safety and high controllability.^4^

On the other hand, localization methods^5,6^ and medical imaging techniques^7^ should be used for monitoring MRs inside the body. Different imaging modalities, including traditional techniques such as ultrasound (US),^8^ magnetic resonance imaging,^9^ single photon emission computed tomography,^10^ and x rays^11^ as well as more recently developed techniques, such as photoacoustic tomography,^12^ have been used for visualizing and tracking MRs in simulated body environments. Each technique comes with its advantages and disadvantages, and identifying the most appropriate imaging modality is often application dependent.^13^ In this complex panorama, US proved to be a mature technology combining decent spatial resolution, deep penetration, and real-time operation with low cost equipment and no harmful radiations.^14^ This makes it one of the best candidates for MRs monitoring and controlling in diverse body districts.

However, US imaging of medical MRs inside the body still presents several challenges. The modalities based on the intensity of backscattered US waves, such as the widely adopted brightness (B)-mode, show poor performance when tracking MRs in tissues. In fact, heterogeneous biological tissues feature high echogenicity (i.e., reflectivity to US waves) and produce high contrast imaging artifacts that might limit MRs visibility and hamper tracking.^15^ To enhance MR visibility, contrast agents, such as microbubbles, could be used.^16^ However, they suffer from short lifespan (10–15 min),^17^ and the resulting contrast might be insufficient due to the small number of microbubbles (diameters of around 10 *μ*m) that might be realistically integrated on-board MRs.

A possible strategy to improve MRs contrast to US exploits MRs motions, typically generated during navigation and task performance. One widespread US imaging technique for motion visualization is color Doppler, generally used in the clinical practice to characterize blood flow velocities. Lately, color Doppler imaging has been successfully used for imaging weakly reflective MRs in controlled experimental conditions by visualizing the relative displacements produced in the surrounding medium.^18,19^ Acoustic phase analysis (APA) of US raw radio frequency (RF) data has been proposed more recently to provide a selective detection of the MR motions,^20^ rather than the detection of relative displacements in the medium. In fact, accessing RF data allows one to detect and process signal features that are not directly accessible from beamformed and reconstructed US images.^21,22^ This approach offers a unique advantage: characteristic MR motions can be filtered out from background motions, thus enabling targeted MR contrast-enhancement even in high echogenic and dynamic conditions (e.g., in-body environments). However, this approach relies on controlling the MR motion features and directions in order to maximize acoustic phase modulation for optimal imaging conditions. For these reasons, it has only been demonstrated with relatively simple MR locomotion schemes, which exhibit in-plane motions with respect to the imaging setup, such as axial vibrations or in-plane rotations.^23^ To progress toward more relevant medical microrobotic scenarios, extending such a technique to a wider repertoire of motion schemes is necessary, possibly including out-of-plane motion patterns such as those of commonly adopted MRs (i.e., helical propellers^24–26^).

To fill this gap, in the present study, we combine a kinematic analysis of the out-of-plane motions of helical MRs with specific US-APA imaging for precise and robust detection and tracking in bio-mimicking environments, where traditional US imaging techniques result unsuitable. To validate the proposed approach, we performed navigation and tracking of a magnetic helical MR in tissue-mimicking phantoms and chicken breast tissues. Moreover, we compared the results obtained with the proposed technique to those obtained with traditional color Doppler imaging, considered as the gold standard motion-based US imaging modality. To assess the robustness of our technique, we also tested it in the presence of diverse background motion disturbances, which are very likely to happen in a realistic *in vivo* scenario.

The remainder of this paper is organized as follows: Section II reports and discusses the set of experiments performed to evaluate the efficiency and robustness of the proposed method in comparison to traditional Doppler during MR tracking in different simulated in-body conditions. Sec. III draws the conclusions for this study and envisions possible perspectives. Sec. IV describes the physical principles behind the motion-induced acoustic phase modulation effect and provides insight into the kinematic model of the helical propeller with a particular focus on its interaction with the US waves during locomotion. This section also illustrates the employed US image processing algorithms and presents the experimental validation platform.

## II. RESULTS

We designed a set of experiments to analyze the performance of the proposed US-APA strategy for imaging and tracking helical propellers in simulated body environments. Different experiments were conducted to assess the motion-enabled contrast-enhancement mechanism, the signal to noise ratio (SNR), the tracking accuracy, and the robustness to possible environmental disturbances that might occur *in vivo*. In all experiments, the performance of US-APA was compared to that of color Doppler imaging, considered as gold standard motion-based US imaging modality.

### A. Microrobot-generated motion signal during locomotion in a straight lumen

**FIG. 1.**
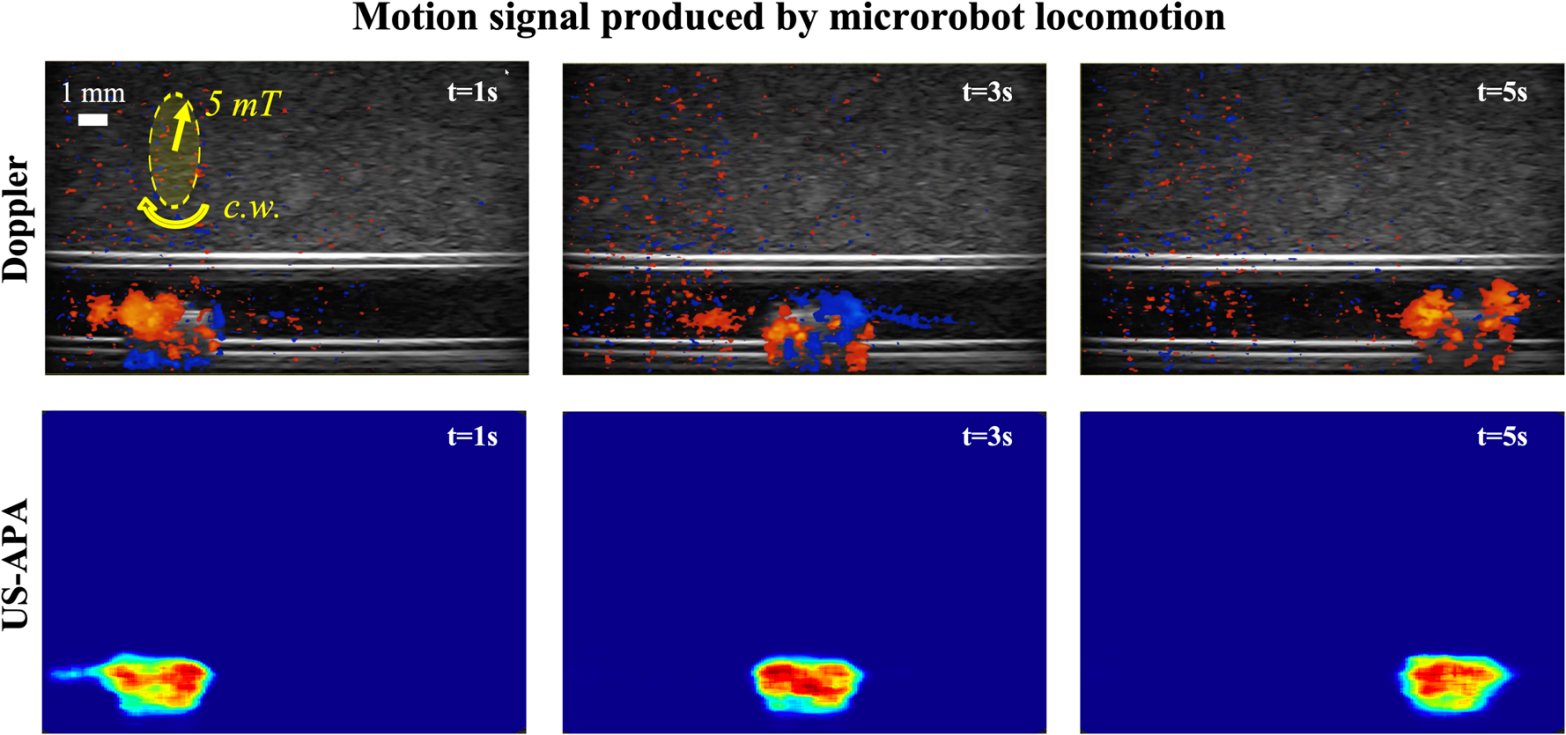
Imaging experiments during locomotion in a straight lumen. Yellow arrows represent the field intensity and rotation direction (c.w. stands for clockwise). The motion signals obtained with color Doppler and US-APA images of the same region are compared at different time points along the MR trajectory. The specific helical propeller motions successfully enhance its contrast in US-APA images with respect to other high echogenic elements (e.g., white lumen boundaries in Doppler images).

As a first step, we qualitatively assessed the MR motion signal during locomotion across a straight lumen in the tissue-mimicking phantom using both Doppler imaging and US-APA (Fig. 1). The MR was actuated with constant rotating magnetic field (frequency of 4 Hz) in static fluid. The rotation of the MR blades produced a constant forward velocity that made the MR swim through the lumen. The collected color Doppler images showed that the rotation of MR blades effectively produced some signal even in the MR long axis view configuration. This result validated the assumptions made in Sec. IV B, regarding the effects of the imaging plane physical thickness. However, the signal intensity was quite weak. To augment the MR-produced Doppler signal, the digital gain needed to be increased, leading to an amplification of noise (Fig. 1, Doppler images, color signal in regions outside the MR body) and a general degradation of the signal to noise ratio (SNR). As a consequence, the Doppler signal distribution was quite random with signal spots also in regions outside the MR body.

On the other hand, the helical propeller in US-APA images showed very good contrast with respect to the background. In fact, different from color Doppler imaging, the US-APA signal was only concentrated within a limited region, as large as the MR body, and null everywhere else in the image, demonstrating the background noise rejection features of US-APA. Not only this characteristic leads to a better SNR and a more stable MR detection, but also it provides a more precise localization of the signal source (i.e., the MR). For more details about these experiments, the reader may refer to the supplementary material Video, Part 1.

### B. Microrobot tracking in chicken breast tissue

**FIG. 2.**
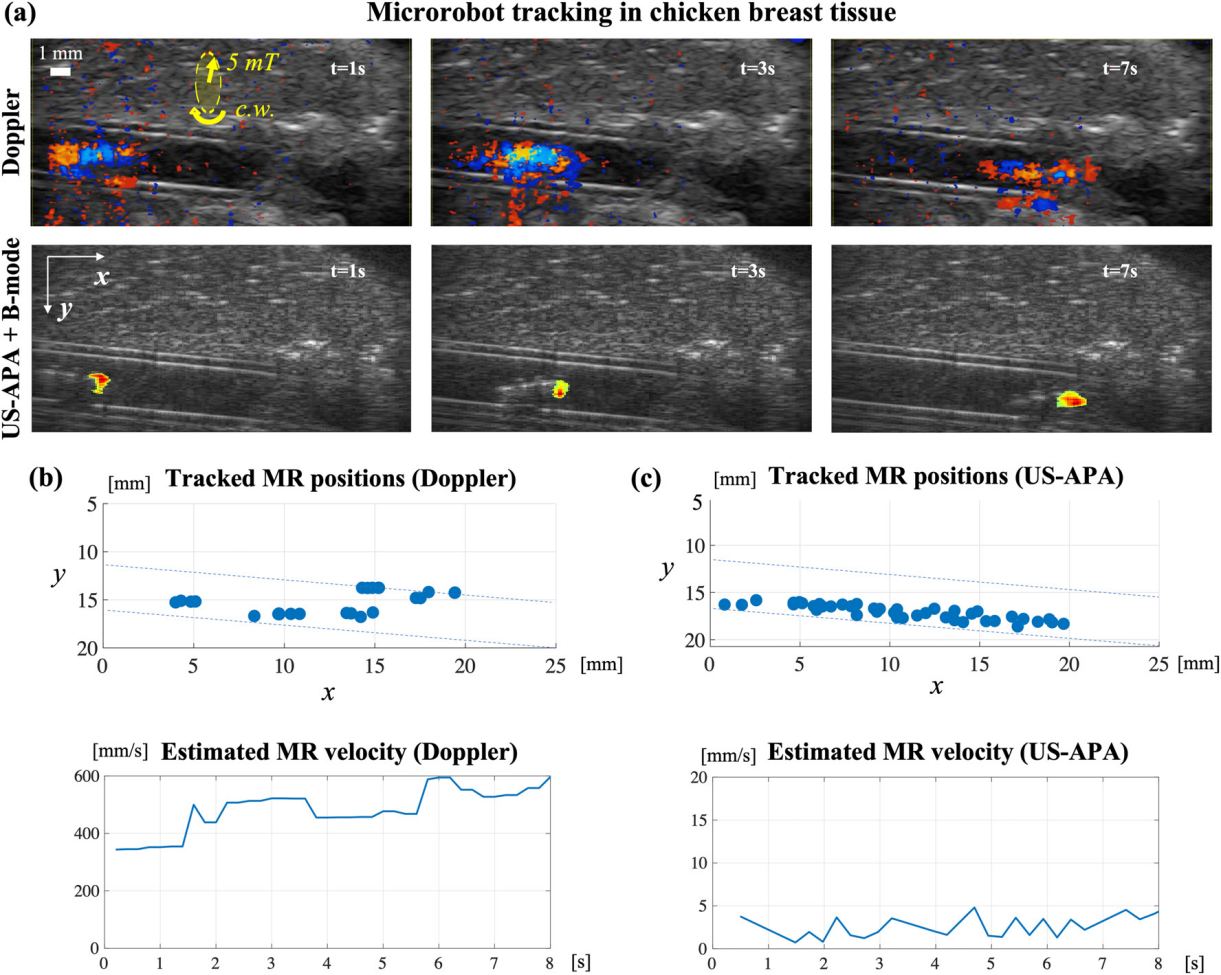
Dynamic tracking experiments in chicken breast tissue. (a) Comparison of images of the same region at different time points along the helical propeller trajectory, obtained with color Doppler and US-APA. Yellow arrows represent the field intensity and rotation direction (c.w. stands for clockwise). (b) and (c) Collection of tracked MR positions and estimated velocity from color Doppler images (b) and US-APA + B-mode images (c). Dashed blue lines represent lumen boundaries.

Once qualitatively evaluated the MR motion signals, we wanted to assess the techniques performance when used for MR dynamic tracking in tissues. For this purpose, we embedded the silicon tube acting as a lumen inside chicken breast tissue with static fluid conditions. The MR was actuated with constant rotating magnetic fields while tracking its position over time with both color Doppler and US-APA (Fig. 2).

At different time instants during locomotion, the results were in line with previous experiments in tissue-mimicking phantom [Fig. 2(a)]. The area around the rotating MR produced Doppler signal, but this signal showed a rather unpredictable pattern being located also outside the MR body. On the other hand, US-APA generated a more predictable and better localized signal with better SNR.

To track the MR trajectory with color Doppler, its centroid was identified by developing a dedicated automatic detection algorithm. MR centroid was assumed as the center of the image region with the highest density of blue and red colors (characteristic Doppler signal) and with the same size as the MR. This allowed to roughly track the MR position over time, but localization precision was poor [Fig. 2(b)]. In fact, by comparing the tracked trajectory with the lumen morphology, visible from B-mode images, we verified that the estimated MR positions in time did not reflect the actual MR trajectory, which instead linearly moved along the lumen bottom boundary [Fig. 2(b), blue dashed lines] due to gravity. The tracking performance further degraded when the MR approached higher echogenic tissue regions [Fig. 2(a), Doppler images, t = 7 s]. Furthermore, when considering the estimated MR Cartesian velocity, obtained by time derivation of the tracked positions, we obtained values around 500 mm/s that were not meaningful from a physical viewpoint. Indeed, the MR traveled 20 mm in around 7 s, resulting in an expected average linear speed of around 2.8 mm/s that is much lower than the measured value. This error was generated by the fact that the estimated MR position was unstable and continuously bounced between pixels relatively far from each other.

On the other hand, US-APA images were analyzed to assess if they could provide better tracking performances. In all the following localization experiments, the US-APA signal intensity for each pixel was normalized with respect to the average spectrum energy for better noise rejection. Furthermore, the US-APA images were hard-thresholded with respect to 50% of the maximum pixel intensity and overlapped on B-mode images (like it is done for traditional color Doppler images), so as to integrate MR specific information from US-APA with morphological and anatomical information on the environment from B-mode [Fig. 2(a), US-APA + B-mode images]. These additional processing steps produced a concentration of the US-APA signal on the propeller front part (with reference to the positive forward velocity), providing better localization of the MR head as well as information regarding the direction of motion. Provided that the MR can be modeled as a rigid body system with known dimensions, the position of the whole body can be derived from the position of the head.

By inspecting the US-APA + B-mode images [Fig. 2(a)], we could assess the stability of the motion signal, which was only present on the helical MR head and null everywhere else in the background. By simply localizing the maximum in US-APA images, we could stably track the MR head positions over time, even when it approached higher echogenic tissue regions [Fig. 2(a), US-APA + B-mode images, t = 7 s]. The tracked trajectory was very close to the lumen morphology, reflecting the expected MR trajectory along the lower boundary. The stable estimation of MR positions over time allowed to precisely measure its Cartesian velocity. In this case, the average velocity measured around 3.2 mm/s that was in line with the real observed MR locomotion features.

Overall, although color Doppler allowed for rough MR tracking, US-APA provided better performance in terms of localization precision, velocity estimation, and tracking stability in echogenic tissues. For more details about these experiments, the reader may refer to the supplementary material Video, Part 2.

### C. Microrobot tracking with motion disturbances

**FIG. 3.**
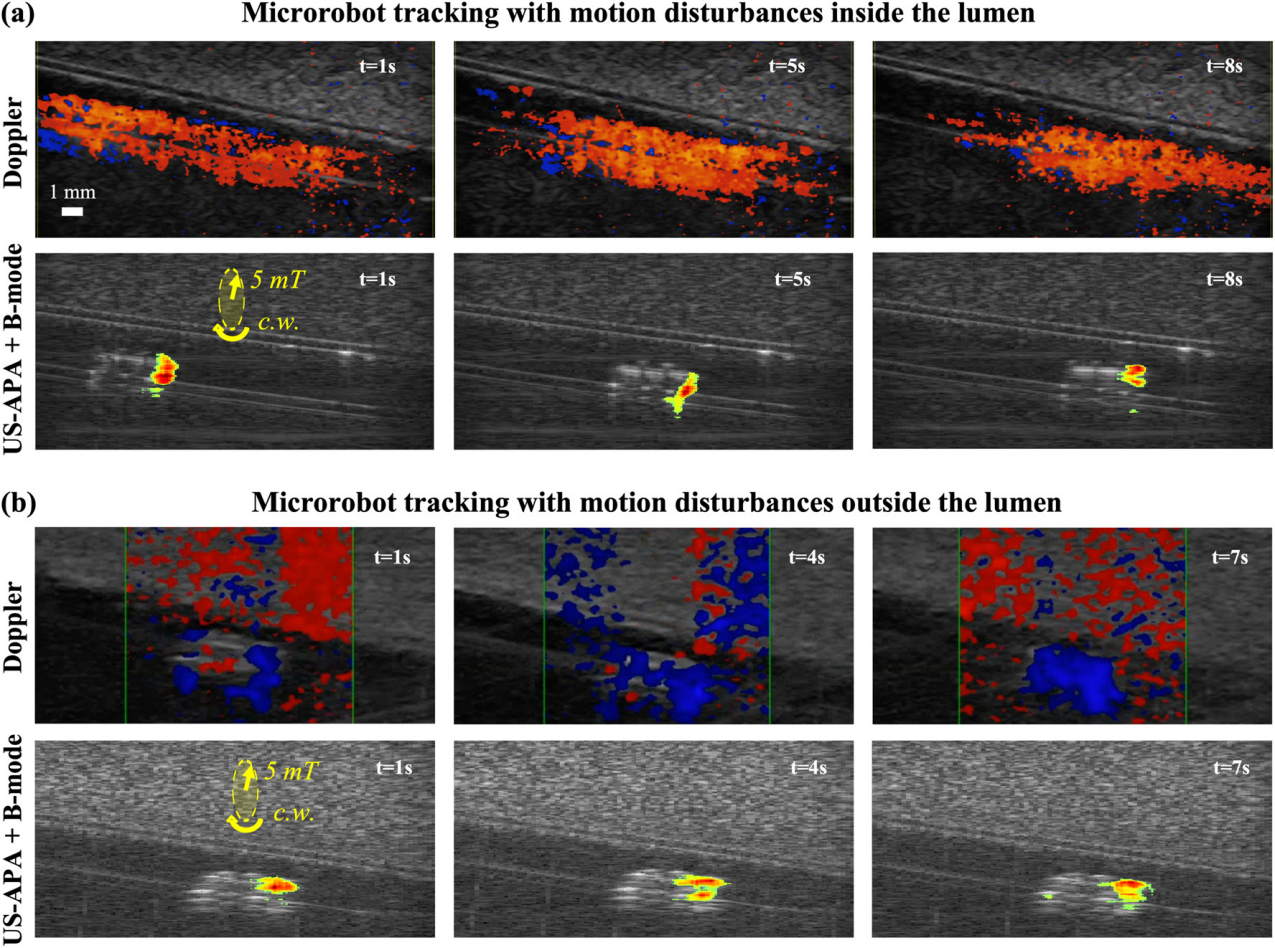
Dynamic tracking experiments in the presence of motion disturbances. Yellow arrows represent the field intensity and rotation direction (c.w. stands for clockwise). (a) Images acquired at different time instants with both color Doppler and US-APA + B-mode during MR navigation against fluid flow in the lumen. (b) Images acquired at different time instants with both color Doppler and US-APA + B-mode during MR locomotion when surrounding tissue motion occurred due to repositioning of the US transducer.

After having assessed the localization performances, we qualitatively evaluated tracking stability and robustness to potential environmental disturbances. Indeed, when targeting *in vivo* applications, different physiological motions, both in the lumina (e.g., fluid flow) and in the surrounding tissues (e.g., due to breathing or heart beating, and US transducer repositioning), could introduce disturbances hampering MR tracking. One possible source of motion disturbance is physiological flow in the lumen. To evaluate its influence, we conducted tracking experiments with the MR moving against counter flow [Fig. 3(a)].

Color Doppler images appeared extremely noisy with motion signal detected in the entire lumen. This prevented from discriminating the signal produced by the helical propeller from the signal produced by the flow, thus making MR localization impossible [Fig. 3(a), Doppler images]. On the other hand, the US-APA signal proved to be robust to the flow disturbance and stably concentrated only on the MR head [Fig. 3(a), US-APA + B-mode images]. In fact, the frequency filtering stage of US-APA imaging (see Sec. IV C) allowed to reject the motions produced by particles in the flow, so as to only visualize the characteristic motion patterns of the target helical propeller.

Aside from the disturbances from fluid flow, the physiological motions of surrounding tissues and those of the actuation/imaging platform could also produce noise signal hampering MR detection. To assess the influence of these motions on the stability of the analyzed techniques, we conducted tracking experiments when the MR was navigated for about 60 mm through the lumen, and the US transducer was continuously repositioned to follow the MR trajectory so as to maintain it inside the imaging plane. This scenario simulated both the possible motions of tissues in the imaging plane and, potentially, the relative motion generated by the adjustment of the US transducer. Color Doppler images showed how the motion of surrounding tissues produced significant signal intensity in the whole region of interest, which again prevented stable MR localization [Fig. 3(b), Doppler images]. On the other hand, when considering US-APA + B-mode images, the motion produced by surrounding tissues could be successfully rejected, allowing to localize the helical propeller as the only source of signal [Fig. 3(b), US-APA + B-mode images].

These experiments showed that color Doppler imaging was significantly affected by motion disturbances that are very likely to happen in a realistic *in vivo* environment. On the other hand, the results of US-APA imaging revealed how its selectivity, based on MR-specific motions detection, could provide a stable MR visualization and localization, even in the presence of other background motion disturbances. For more details about these experiments, the reader may refer to the supplementary material Video, Part 3.

### D. Microrobot tracking in bifurcated lumen

**FIG. 4.**
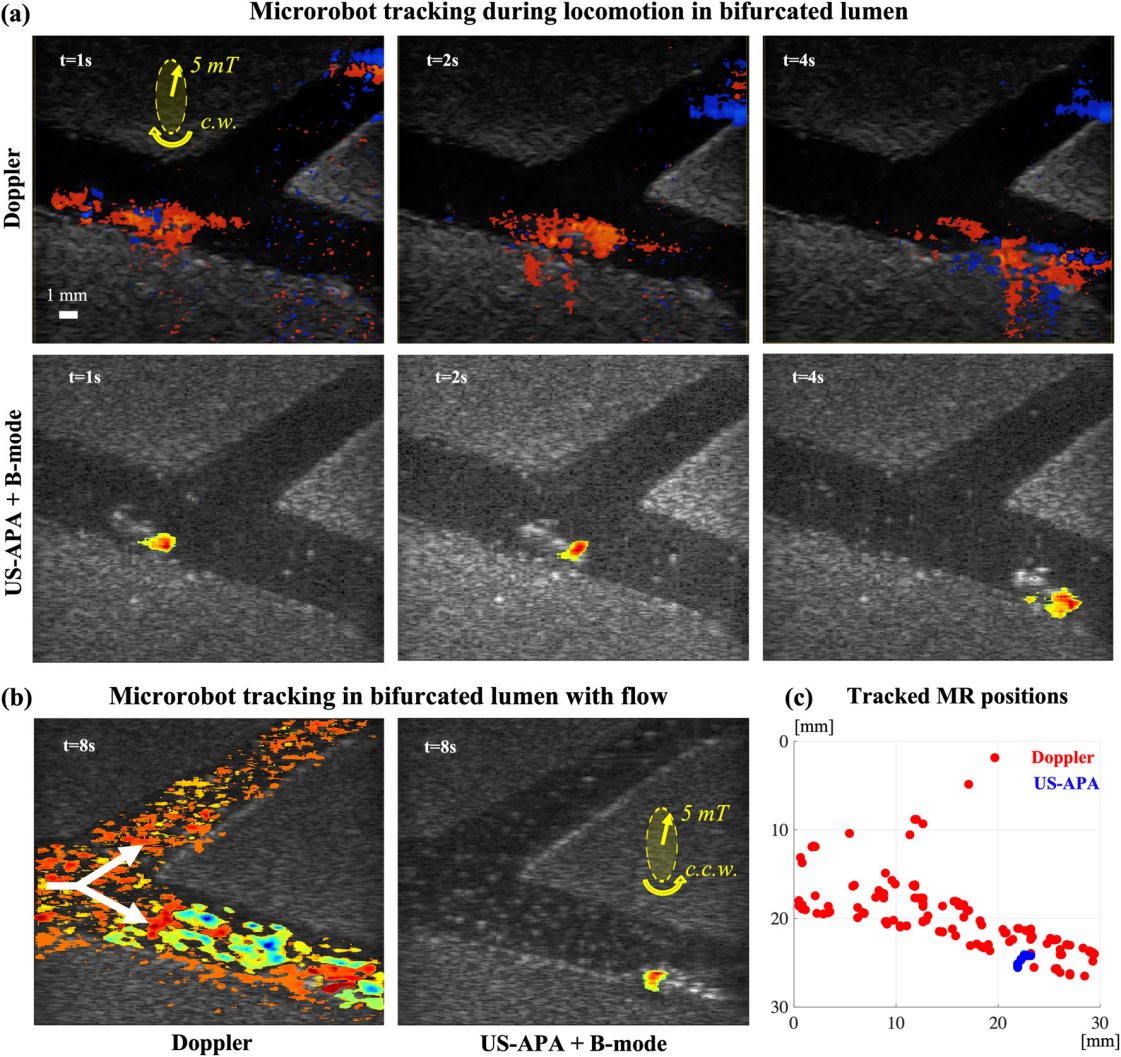
Dynamic tracking experiments in bifurcated lumen. Yellow arrows represent the field intensity and rotation direction (c.w. for clockwise, c.c.w. for counterclockwise). (a) Images acquired at different time instants with both color Doppler and US-APA + B-mode during MR navigation in bifurcated lumen with no pumped fluid flow. (b) Comparison between color Doppler and US-APA + B-mode images after the pumped flow was switched on. The white arrows represent flow directions in the bifurcated lumen. The MR is actuated to swim against the flow so as to stay idle in the target position. (c) Cumulative graph representing the tracked MR positions in the image reference frame using both color Doppler (red) and US-APA (blue).

With the final set of experiments, we wanted to verify the ability of the proposed technique to reliably track the helical propeller in a bifurcated lumen and, consequently, to discriminate in which of the two branches it was located (Fig. 4). First, we navigated the MR through one of the two branches of the bifurcation without any fluid flow to easily reach the target position [Fig. 4(a)]. Although the pump was off, free fluid motion in the bifurcation introduced Doppler signal in the wrong branch, making it hard to discriminate the actual MR path [Fig. 4(a), Doppler images]. This noise signal was rejected in US-APA + B-mode images, which clearly showed the correct MR positions [Fig. 4(a), US-APA + B-mode images]. After reaching the target branch [Fig. 4(b), lower branch], the pumped fluid flow was activated in the bifurcation [Fig. 4(b), white arrows]. At this stage, the MR was actuated in the opposite direction [Fig. 4(b), field directions in yellow] to enable swimming against the flow so as to stay idle in the target position. The MR positions over time were tracked with both color Doppler and US-APA and overlapped on the same graph [Fig. 4(c)].

The data showed how color Doppler resulted in wrong MR localizations around the whole lumen region, caused by flow-generated motion disturbances. In fact, by looking at the tracking data from color Doppler [Fig. 4(c), red data points], it was not possible neither to determine the correct MR position nor in which of the two branches it was located. On the other hand, by looking at US-APA tracking data [Fig. 4(c), blue data points], the MR could be stably localized in the target position in the lower branch.

Overall, US-APA imaging demonstrated to be reliable in tracking the MR in bifurcated lumina, even in the presence of fluid flow, where color Doppler imaging failed due to motion disturbances. For more details about these experiments, the reader may refer to the supplementary material Video, Part 4.

## III. CONCLUSIONS

In this study, we exploited the motions of helical propellers often used in medical exploration as a special signature to enhance their contrast with US-APA, allowing for improved imaging and tracking in echogenic and dynamic tissues. We performed a set of experimental evaluations in which a helical propeller was navigated in diverse simulated *in vivo* scenarios, aimed at reproducing realistic environmental conditions accounting for possible disturbances to MR imaging and tracking. The performance of the proposed technique was compared to those of color Doppler imaging, considered as the gold standard for motion-based imaging of MRs.

Experiments performed in tissue-mimicking phantoms and chicken breast tissues, both in static and dynamic conditions revealed that, although it provided better MR contrast than intensity-based B-mode imaging, color Doppler was sensible to all the motions of the surrounding environment. Other objects moving at higher velocities than the target MR (e.g., particles in the blood flow or motion of surrounding tissues) produced higher Doppler signals, eventually degrading the SNR and hampering MR detection.

Furthermore, even in static background conditions, the unpredictable nature of the MR-generated Doppler signal pattern made it hard to implement a performing automatic detection algorithm and led to poor localization results.

On the other hand, thanks to its MR-specific motion detection, the proposed US-APA imaging strategy provided better SNR and MR tracking performance both in static and dynamic background conditions. In fact, the technique proved robust to different motion disturbances both inside the navigated lumina (i.e., physiological flow) and outside in the surrounding tissues (i.e., physiological tissue motion), paving the way for stable real-time imaging and tracking of MRs in biological environments.

Nevertheless, although it represents advancement with respect to the state-of-the-art, the proposed technique can be further improved. In fact, the US-APA imaging frame rate (2 fps in this study) can be improved by speeding up the MR motion dynamics (e.g., increasing the rotation frequency), but it is also limited by the computational burden of Fourier analysis on hardware.^15^ Future development of this study shall focus on improving the frame rate by implementing faster motion detection strategies, possibly based on more advanced machine learning approaches. Furthermore, additional research effort should be focused on extending the US-APA technique to a larger number of MR motion schemes, including multimodal swimmers^27^ and other promising medical MRs.^28^

## IV. METHODS

### A. Motion-induced acoustic phase modulation

US imaging exploits pulsed pressure waves emitted by a piezoelectric transducer. The direction of waves propagation is called the acoustic axis. When the waves encounter acoustic impedance discontinuities (e.g., interfaces between different materials or physical boundaries), they are partially scattered back toward the source.^29^ Assuming to transmit a sinusoidal pulse, the backscattered echo signal E*(*t*) can be expressed in the following form:

$$E^{*}\left(t\right)=A\left(t\right)\cdot e^{j\varphi \left(t\right)},$$ (1)

where *A*(*t*) is the instantaneous amplitude (i.e., the envelope) and φ(t) is the instantaneous phase. The time variable *t* is the echoes time of flight. It provides information on objects spatial location along the acoustic axis: echoes arriving to the transducer with higher delay (larger *t*) are associated with farther scattering objects.

The signal envelope represents the pressure intensity locally backscattered by objects along the acoustic axis, thus containing information about objects backscattering properties (e.g., their acoustic impedance). In B-mode, *A*(*t*) is converted into brightness levels to create a contrast image of the area exposed to US. Objects with high backscattering properties, for example, heterogeneous tissues,^15^ will result in high intensity (brightness) image areas. In a MR tracking scenario, this implies that if the target MR is close to a highly echogenic object (e.g., the boundary of a lumen), the two entities cannot be distinguished in B-mode images.

On the other hand, the instantaneous acoustic phase φ(t) carries information about the object location with respect to the acoustic axis. Object displacements along the direction of wave propagation linearly modulate the echoes instantaneous phase; therefore, object velocities modulate the acoustic frequency (i.e., the time derivative of the acoustic phase). This effect extends to displacements along any direction by considering their projection along the acoustic axis. The phase information is unrelated to the relative echoes intensity and, thus, to the objects backscattering properties. For this reason, MRs motion can be used to enhance their contrast even in highly echogenic backgrounds. This principle is typically exploited in clinical Doppler imaging to characterize blood flow velocities. However, by accessing US raw data, it can be further exploited to perform specific acoustic phase analysis and detect the characteristic motion features of helical propellers.

### B. Kinematic model and characteristic motions of helical propellers

**FIG. 5.**
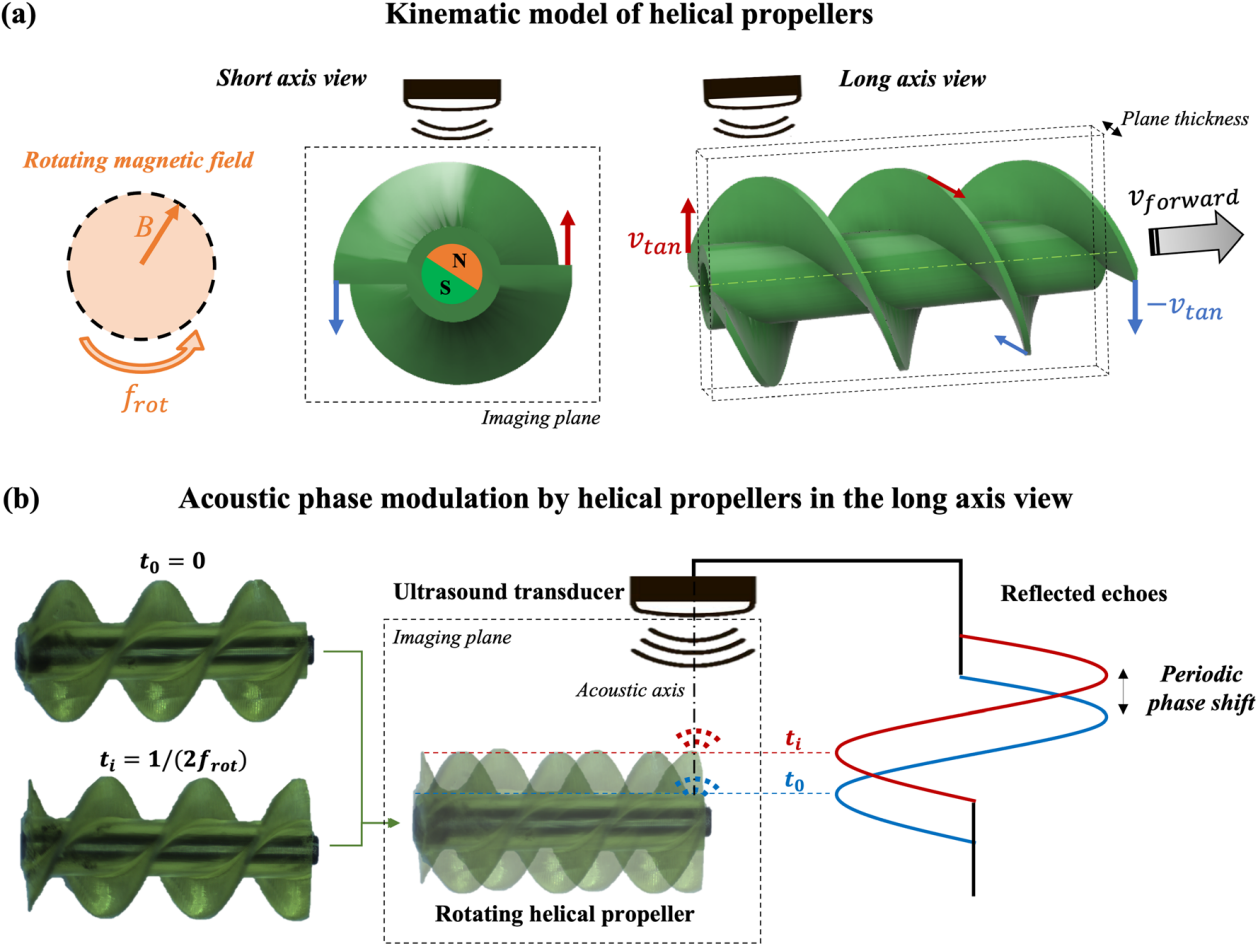
Helical propellers motion and acoustic phase modulation. (a) Schematic representation of the relative velocities on the MR blades extremities under a rotating magnetic field. Both the propeller short axis and long axis views are reported. The green dashed line represents the MR rotation axis (i.e., the long axis). (b) Specific modulation of the acoustic phase produced by the helical propeller blades motion projected in the long axis view. The blades' profile projected in the long axis view evolves in time like a sinusoidal traveling wave. This motion produces harmonic phase shifts in the waves backscattered by each fixed point of the helical structure.

Helical propellers are one of the most adopted MR designs. Inspired by bacterial flagella, these MRs can achieve very efficient and controllable 3D locomotion at low Reynolds numbers, converting rotational motion into linear. By providing the helical structure with radial magnetization, MR rotation around its long axis can be induced by applying a rotating magnetic field [Fig. 5(a)]. During rotation, the helical blades interact with the surrounding medium, generating drag forces that produce a forward velocity parallel to the MR axis of rotation.^30^ From a kinematic viewpoint, if a helical propeller with radius $r_{MR}$ rotates with frequency $f_{\mathrm{rot}}$, the extreme points of its blades feature a relative tangential velocity $v_{\;\text{tan}\;}=2\pi r_{MR}f_{\mathrm{rot}}$. Independent of the direction of rotation, the extremities of the structure (left and right) have opposite velocity directions with respect to the rotation axis [Fig. 5(a)]. The resulting propeller forward velocity $v_{\mathrm{forward}}$ is linearly related to its rotation frequency $f_{\mathrm{rot}}$, if not considering any turbulence.^31^

When imaging helical propellers with 2D US, the imaging plane can be placed to enable either MR short axis or long axis views [Fig. 5(a)]. The first view [Fig. 5(a), short axis view] provides the advantage that the blades' tangential velocities [red and blue arrows in Fig. 5(a)] are parallel to the acoustic axis, thus producing an optimal modulation of the acoustic phase (see Sec. IV A). This effect has been successfully exploited to visualize the helical propeller velocity distribution in color Doppler images.^32^ However, in this configuration, the MR forward velocity would be orthogonal to the imaging plane. This implies that MR tracking requires a highly reactive visual-servoing control system able to quickly reposition the imaging plane (i.e., the US transducer) to follow MR locomotion. The implementation of such a tracking system is not straightforward, and very likely, it would not be robust to disturbances dragging the MR out from the imaging plane.

A more convenient solution would be to choose the optimal imaging plane-parallel to the MR long-axis [Fig. 5(a), long axis view]. In this configuration, the MR forward velocity would always lay within the imaging plane, allowing a more stable dynamic tracking. However, this implies also having the MR axis of rotation [i.e., the MR long axis, green dashed line in Fig. 5(a)], laying in the same imaging plane. Theoretically, in this configuration, the rotating blades tangential velocities [red and blue arrows in Fig. 5(a)] have always null projection on the acoustic axis, thus not producing any modulation of the acoustic phase nor Doppler signal. In the reality, the imaging plane of a US transducer features a non-null physical extension [plane thickness in Fig. 5(a)] in the out-of-plane direction due to the intrinsic width of the US beam. Furthermore, the plane would unlikely be perfectly aligned to the MR long axis [green dashed line in Fig. 5(a)]. For these reasons, as long as the imaging plane intersects the MR body, there will always be a fraction of the rotating blades having a projected motion along the acoustic axis and actively modulating the acoustic phase signal. However, due to the uncertain arrangement of the imaging plane with respect to the MR long axis, the produced Doppler signal is expected to be rather unpredictable and, thus, hard to recognize in a dynamic environment. For this reason, a more predictable MR motion signal pattern should be identified in order to acquire stable feedback through specific US-APA.

Based on these considerations, the projected motions of the rotating MR blades in the long axis view were considered [Fig. 5(b)]. Such a motion pattern resembles that of traveling waves, where the peaks in the blades sinusoidal spatial profile move parallel to the MR long axis. For example, the blade profiles at different time instants $t_{0}=0$ and $t_{i}=1/(2f_{\mathrm{rot}})$ look like two sinusoids delayed by π/2 [Fig. 5(b), rotating helical propeller]. Therefore, during MR locomotion, the echoes reflected by fixed points on the MR body periodically assume different positions along the acoustic axis [Fig. 5(B), reflected echoes for $t_{0}$ and $t_{i}$]. When the MR rotates with constant frequency, this projected motion pattern of the blades produces periodic phase shifts in the MR backscattered waves, having the same frequency as the MR rotation. These periodic phase shifts can be isolated by Fourier analysis and exploited as a specific motion signature, allowing to selectively enhance the MR contrast even in complex and dynamic environments.

### C. Image processing algorithms

**FIG. 6.**
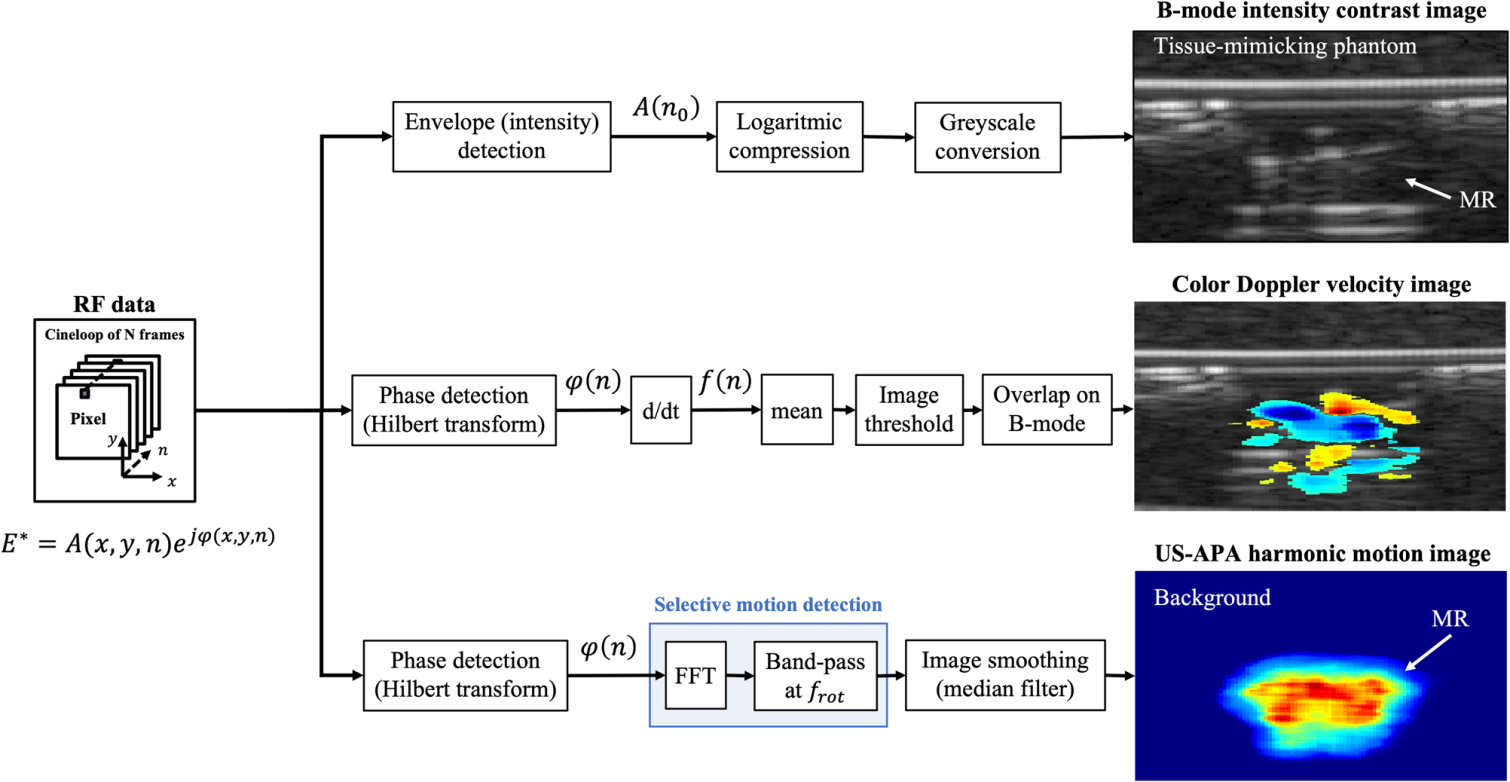
Schematic representation of the signal processing steps involved in the generation of US images of the rotating helical propeller according to the three different modalities. B-mode contrast images are elaborated from the echoes acoustic intensity. Color Doppler velocity images are elaborated from the echoes acoustic frequency. US-APA harmonic motion images are elaborated from the echoes acoustic phase after selective motion filtering in the frequency domain.

The specific periodic motion pattern of helical propellers can be exploited to develop a custom US-APA imaging modality allowing to enhance their visibility and to provide better contrast with respect to traditional US imaging techniques such as B-mode and color Doppler (Fig. 6).

When considering a cineloop (i.e., an ensemble of *N* RF data frames), for each pixel in the frame coordinate system (x, y), the echo analytic signal in the frame temporal dimension (*n*) is given in Eq. (1). To produce a B-mode image, the signal amplitude $A(n_{0})$ relative to the first frame is extracted by envelope detection. The envelope values are logarithmically compressed and converted into grayscale levels to produce B-mode images, representing objects echogenicity. Although the MR is poorly visible in B-mode images (Fig. 6, B-mode image), they provide important morphological and anatomical information on the surrounding environment (e.g., tissues, lumen boundaries, and obstacles).

On the other hand, to extract motion information, the signal phase φ(n) is detected by Hilbert transform. To produce a color Doppler image, the phase is time derived to obtain the acoustic frequency f(n), which is then time averaged to obtain the mean velocity distribution within the cineloop time frame. In traditional color Doppler, this velocity image is hard-thresholded with respect to 50% of the maximum pixel value and finally overlapped on a B-mode image. The color Doppler images represent the velocities of all objects in the imaging plane (Fig. 6, color Doppler image).

To perform a more selective and MR-specific motion detection, the periodic patterns of the MR rotating blades can be better detected with US-APA harmonic motion images.^15^ To this purpose, the phase φ(n) is analyzed in the frequency domain by the fast Fourier transform (FFT) and band-passed at the blades rotation frequency $f_{\mathrm{rot}}$. This can be done by means of the computationally efficient Goertzel algorithm.^33^ This harmonic motion image is then smoothed by means of a 2D median filter with kernel of 1 × 1 mm^2^, so as to remove outliers. The final US-APA images represent the intensity of harmonic motions with frequency $f_{\mathrm{rot}}$ (Fig. 6, US-APA image). As long as the MR is the only object in the imaging plane performing such periodic motion patterns, this imaging modality provides a powerful MR-specific contrast-enhancement mechanism.

### D. Experimental validation platform

**FIG. 7.**
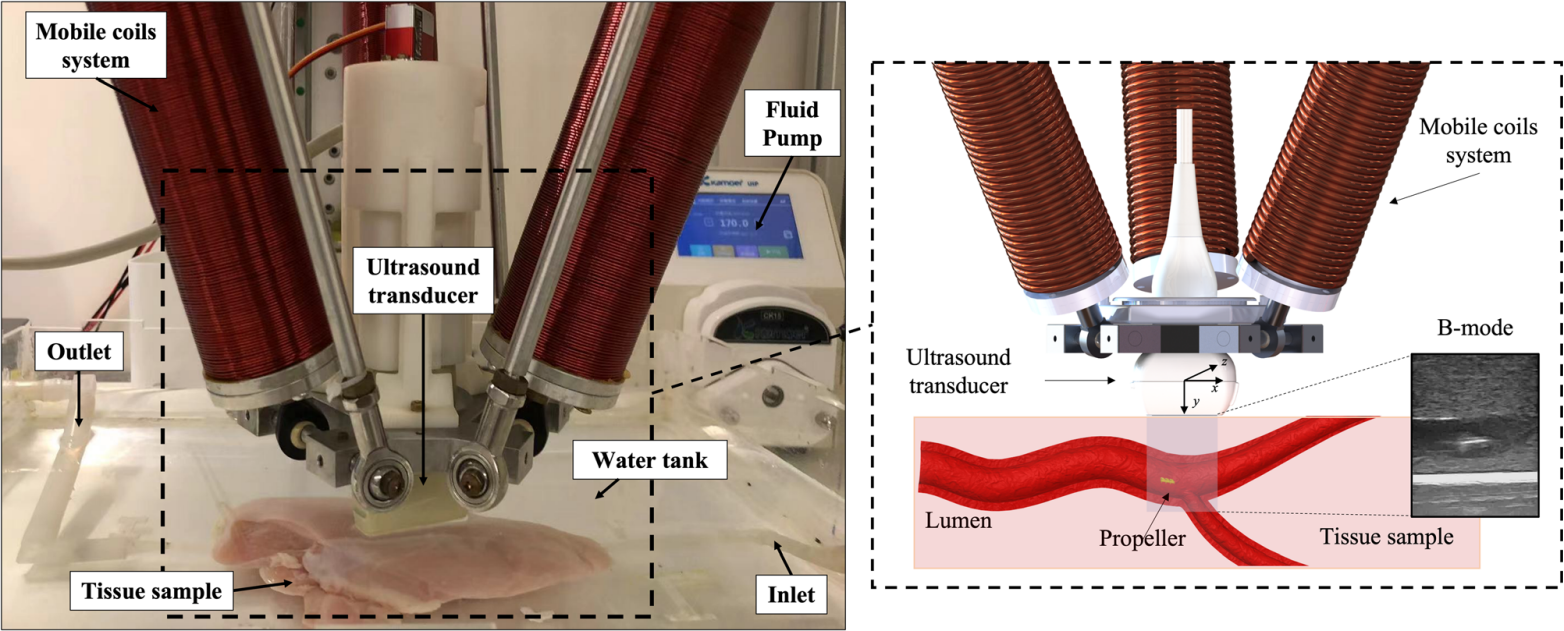
Experimental platform for MR navigation and imaging in simulated *in vivo* conditions. The black dashed box on the right represents a schematic detailed view on the arrangement among the mobile coils system, the ultrasound transducer, and the tissue sample.

To validate the proposed MR imaging strategy, we developed an experimental platform able to simulate MR navigation in realistic *in vivo* environments, accounting also for possible disturbances produced by tissue echogenicity and physiological motions. To this purpose, the platform included a tissue-mimicking phantom with artificial lumina filled by blood-mimicking fluid, a fluidic pump, a magnetic helical MR placed inside the phantom lumen, a remote magnetic actuation system (mobile coils system), and a US imaging apparatus (US transducer) (Fig. 7).

Phantoms were designed to mimic a tract of body lumen (4 mm in diameter) with the surrounding soft tissues. To simulate possible heterogeneity and high contrast regions in actual human soft tissues, agarose was doped with soy milk used as a scatter-enhancing agent. Agarose powder (Sigma-Aldrich) was dissolved in a de-ionized and degassed water (dd-H_2_O)—soy milk (5% v/v) solution and kept at 90 °C for 1 h under continuous stirring. Agarose concentration (2% v/v) was selected to mimic human tissues mechanical and acoustic properties.^34^ Pre-shaped 4 mm diameter silicone tubes were embedded in the phantom before reticulation to act as lumina. The US reflectivity of silicone tubes walls simulated that of natural interfaces at the tissue-lumen boundaries. Physical reticulation occurred at room temperature in the target mold (4.5 × 4.5 × 20 cm^3^). A fluid that mimics the blood in terms of density (1006 Kg/m^3^), viscosity [0.004 Kg/(m s)], and acoustic properties was obtained from an aqueous glycerol solution (60% v/v).^35^ Cellulose powder was added to the aqueous solution (0.7 mg/ml) as a scatter-enhancing agent. Fluid flow was generated in the lumina by means of a peristaltic pump (Kamoer, UIP CK15, China). The average flow rate in a 4 mm diameter lumina was 4.86 cm/s. This value is close to physiological flow values in medium arteries.^36^ The considered experimental conditions correspond to a Reynolds number of 51.5, which results in laminar flow in the lumen.

The helical propeller was 3D printed with polyethylene glycol diacrylate (BMF Precision, China) using projection microstereolithography (NanoArch S130, BMF Precision, China). It had a diameter of 2.3 mm and a length of 5 mm with an internal 1 mm channel for magnet lodgment. After printing, the MR was dried in oven for 1 h. After that, a cylindrical NdFeB permanent magnet with a diameter of 500 *μ*m and a length of 5 mm was embedded in the long axis channel. The magnetic field on the surface of the NdFeB permanent magnet measured around 72 mT.

The magnetic MR was actuated by a rotating magnetic field, produced by a mobile three coils system,^37^ positioned above the tissue-mimicking phantom. In all experiments, the average magnetic field intensity in the coils workspace was 5 mT, and the rotation frequency was 4 Hz. The US imaging transducer (Telemed, L18-7H30-A5, Lithuania) was fixed inside the mobile coil system with an eye-in-hand approach so that the imaging plane always laid within the magnetic manipulation workspace and moved rigidly with the coils system (Fig. 7). The US transducer was always in contact with the phantom for optimal acoustic coupling, and the maximum imaging depth was 4 cm. An open architecture digital beamformer (Telemed, ArtUs, Lithuania) provided clinical US imaging modalities (e.g., B-mode and color Doppler) as well as real-time access to raw RF data, allowing for custom image processing (e.g., US-APA). The B-mode and color Doppler frame rates were, respectively, 80 and 30 fps. Since the US-APA frame rate is directly related to the MR motion dynamics,^15^ a MR rotation frequency of 4 Hz resulted in a US-APA frame rate of 2 fps.

## SUPPLEMENTARY MATERIAL

See the supplementary material for videos regarding the MR tracking experiments in tissue-mimicking phantoms and chicken breast tissues.

## Data Availability

The data that support the findings of this study are available from the corresponding authors upon reasonable request.
